# Low sensitivity of needle aspiration cultures in patients with cellulitis/erysipelas

**DOI:** 10.1186/s40064-016-3293-z

**Published:** 2016-09-15

**Authors:** Rein Jan Piso, R. Pop, M. Wieland, I. Griesshammer, M. Urfer, U. Schibli, S. Bassetti

**Affiliations:** 1 Medizinische Klinik, Kantonsspital, Baslerstrasse 150, 4600 Olten, CH Switzerland; 2 Bakteriologisches Institut Olten, Kantonsspital, Olten, Switzerland; 3 Division of Internal Medicine, University Hospital Basel, Basel, Switzerland

## Abstract

**Purpose:**

Cellulitis is normally treated without knowledge of the responsible pathogen. Blood cultures are positive in about 2–4 %, and superficial swabs are of no value. Needle aspiration has been proposed with identifying the likely pathogen in up to 29 %, but these studies are of older date and the technique is not widely used.

**Methods:**

We prospectively evaluated the sensitivity of needle aspiration cultures in all patients with erysipelas/cellulitis. Diagnosis was made clinically by the treating physician. Needle aspiration was done with a 1 ml syringe and a 26G needle. The needle was removed and the syringe brought to the microbiological laboratory and analysed according to standard procedures.

**Results:**

95 Patients were seen during a period of 22 month. 4 Patients were excluded, as diagnosis was not confirmed. Cellulitis was present in 10/91 and erysipelas in 81/91 patients. In the first 25 patients with needle aspiration from the margin, none was positive. In 8/66 (12 %) patients where needle aspiration was done at the site of maximum inflammation, the pathogen was identified. 4/8 Cultures were positive for S. aureus, 2/8 for streptococci and 2/8 for other bacteria. In 11/66 (16.6 %) patients, skin colonisation flora was detected. In the subgroup of patients without prior antibiotic treatment and needle aspiration from the site of maximum inflammation, sensitivity was slightly better 8/55 (14.5 %; 95 % CI 7.5–25.8 %).

**Conclusions:**

Needle aspiration culture had a low sensitivity for detecting responsible pathogen in patients with cellulitis/erysipelas. No impact in antibiotic treatment could be observed.

## Background

Skin and soft tissue infections are a significant problem in health care facilities. A marked increase could be observed between 1993 and 2005 (Pallin et al. 2008). This trend continues with an increase of 9 % of patients seen in the emergency department between 2006 and 2011 (Weiss et al. 2014), cellulitis being the fifth most common diagnosis in ED (Weiss et al. 2011).

Almost all patients are treated empirically (Garau et al. 2015). While the possible spectrum of causative pathogens is relatively large, the vast majority of infections are due to staphylococci and streptococci (Moet et al. 2007). Streptococci are still sensitive to penicillin, but increasing numbers of staphylococci are not sensitive to betalactam-antibiotics (Benoit et al. 2014; Edelsberg et al. 2014). While in Switzerland Amoxicillin-clavulanate acid is the most frequent drug used empirically, the spectrum of prescribed antibiotics is relatively large including coverage of methicillin resistant staphylococci, depending on local epidemiology (Garau et al. 2015).

As the prevalence of resistant pathogens differs considerably between different regions, global or continental wide guidelines may not offer best treatment options for all patients (Johnson 2011). Knowledge of the causative pathogen could have an important impact in treatment outcome and limit inadequate antibiotic prescriptions. However, blood culture is only positive in 2–8 % of skin and soft tissue infections (Perl et al. 1999; Gunderson and Martinello 2012) and skin swabs are not reliable in detecting the pathogen responsible for the infection (Mandell et al. 2010).

Needle aspiration has be proposed in a small number of studies, but these included often only a small number of patients and are of older date(Newell and Norden 1988; Sachs 1990; Patel Wylie et al. 2011; Lebre et al. 1996; Sigurdsson and Gudmundsson 1989). As these studies gave conflicting results, needle aspiration has not become part of standard diagnostic practise in patients presenting with skin and soft tissue infections. We decided to perform needle aspiration in all patients presenting with cellulitis or erysipelas at our hospital and monitor the sensitivity and clinical impact of the technique.

## Methods

From November 2011 until November 2014, needle aspiration was performed in all patients admitted with clinical diagnosis of erysipelas or cellulitis to the emergency department of the Kantonsspital Olten, a 300 bed university affiliated teaching hospital.

The local ethic committee approved the study and waived the requirement of a written informed consent, since needle aspiration is an established diagnostic procedure without known complications, causing minimal discomfort, if any, to the patient. All patients consecutively admitted to the emergency department of our hospital were included, with exception of those in whom inclusion was missed at entrance and antibiotic treatment was started before the needle aspiration was performed. Prior outpatient treatment with oral antibiotics was not an exclusion criterion as the treatment seemed to fail, but these patients were analysed separately.

Diagnosis of cellulitis/erysipelas was made clinically by the treating physician. An experienced infectious diseases specialist visited all patients within 48 h to confirm the diagnosis and to evaluate the antibiotic treatment.

Needle aspiration was done before introduction of antibiotic treatment from the margin of the inflammation in the first 25 patients, but then changed to the point of maximal inflammation. With a BDPlastikpak™ Luer 1 ml syringe and a BD Microlane™3 26G ½” needle 0.5 ml sterile saline 0.9 % was injected intradermal and subsequently directly aspirated. The needle was removed and the syringe capped and brought directly to the microbiological laboratory, where the aspirate was cultivated on a McConkey agar plate, according to standard procedures. Two pairs of blood cultures were taken from every patient.

The patients were treated according to local antibiotic guidelines, mainly with amoxicillin/clavulanate. During winter 2014/2015, due to a European wide shortage of amoxicillin/clavulanate, recommendation was changed to cefazolin as empiric treatment for hospitalized patients.

## Results

95 Patients were seen during a period of 36 month. 4 patients were excluded, as diagnosis was not confirmed. In 10/91 patients, cellulitis was diagnosed, 81/91 patients presented with an erysipelas. In the first 25 patients where needle aspiration was performed from the margin of the redness, none was positive. Therefore we changed the procedure and performed the aspiration at the side of the maximal inflammation. In 8/66 (12 %) patients where needle aspiration was done at the site of maximum inflammation, the causative pathogen was identified by needle aspiration. 4/8 (50 %) cultures were positive for S. aureus, 2/8 (25 %) for streptococci and 2/8 (25 %) for other bacteria (1 Enterobacteriaceae sp, 1 Enterococcus sp). In 11/66 (16.6 %) patients, skin colonisation flora was detected by aspirate. [*S. epidermidis*(8), *P. acne*(2), *Corynebacterium* sp(1)]. 11 Patients had prior antibiotic treatment. Sensitivity for erysipelas was not different from cellulitis (6/81 vs 2/10 *p* = 0.21). Neither blood culture nor needle aspirates cultures were positive for likely pathogens in these patients. In the subgroup of patients without prior antibiotic treatment and needle aspiration from the site of maximum inflammation, sensitivity was slightly better 8/55 (14.5 %). In 2/66 patients with negative needle aspirate cultures, blood cultures were positive (*S. aureus*). Empirical antibiotic treatment was not changed in any patient due to results of needle aspiration. See Table 1 for results.

**Table 1:** Sensitivity on needle aspiration in cellulitis/erysipelas

	Positive cultures/number of patients	Sensitivity (%)	CI 95 %
Aspiration from margin *All patients*	0/25	0	0–13
Aspiration from centre *All patients*	8/66	12	6–22
Aspiration from the centre *Patients without prior AB therapy*	8/55	14.5	7.5–25
Contamination *Aspiration from the centre*	11/66	16.6	9.6–27

## Discussion

An easy to perform, minimal invasive diagnostic technique for the identification of the causative pathogen of cellulitis and erysipelas, such as needle aspiration cultures, would be very helpful, particularly in view of the increasing incidence of skin and soft tissue infections and considering the spread of resistant bacteria such as community-associated methicillin resistant S. aureus. However, information on how to perform needle aspiration and on its clinical utility is rather scarce and conflicting. As some authors favour the margin of the lesion as best localisation to perform needle aspiration (Sachs 1990; Epperly 1986), we began study with this technique. However, the results were disappointing, as none of the 25 aspirates showed a positive result. The subsequent change to the site of maximal inflammation yielded somewhat better results. However, sensitivity did not exceed 15 % even for those patients who did not have prior antibiotic treatment. The rate of positive needle aspirate cultures in our study is congruent to some earlier studies (Newell and Norden 1988; Patel Wylie et al. 2011; Epperly 1986; Goldgeier 1983; Howe et al. 1987), while others reported positive results in up to 36.4 % (Sigurdsson and Gudmundsson 1989) and even 81 % (Lee et al. 1985). In this study by Lee et al., only 21 patients with cellulitis were studied and in those without antibiotic treatment, 9/11 (81 %) had positive results. The authors immediately suspended the aspirate in 0.2 ml saline and inoculated the suspension directly in the microbiological laboratory. We also tried to perform the microbiological cultures as soon as possible after aspiration, however, the delay may have been larger, as the aspirates were transported to the microbiological laboratory with the other routine microbiological specimens and processed only during standard working times (8 am–5 pm, and 8 am–12 am during holidays) Another difference was the technique used for aspiration. We performed a technique of aspiration used in most studies injecting 0.5 ml sterile saline before aspiration (Newell and Norden 1988; Sachs 1990; Lebre et al. 1996; Epperly 1986). Lee et al. (1985) did not inject a small amount of saline but used an empty syringe moving forward and backward the needle in different directions, comparable to puncture for cytological evaluation. However, the high sensitivity found by Lee et al. may be related to differences in the study populations. A substantial number of Lee’s patients needed surgical intervention during the hospitalisation. Therefore this study population may not have represented the usual patients with cellulitis or erysipelas. In our study the aspiration was performed by different attending physicians of the emergency department. Even if these physicians were trained in the technique, the number of aspirations performed by each physician was relatively low. Better result may be obtained if only a few, well trained persons perform the aspirations. We did not find a difference in sensitivity between erysipelas and cellulitis. As cellulitis involves subcutaneous tissue, intradermal aspiration may not be the best method. However, as the technique is rather demanding and clinical differentiation between cellulitis and erysipelas sometimes difficult, we do not think differentiated aspiration technique would have enhanced sensitivity.

There is one study using ultrasound technique in needle aspiration for finding the best area to collect pathogens (Noh et al. 2011). However, even if sensitivity rate of 73.9 % was achieved, this technique is not validated, time consuming and requests further training for the performing physician.

## Conclusion

Culture of needle aspirates performed as routine diagnostic procedure in patients with cellulitis or erysipelas had low sensitivity and no impact on patient management. If needle aspirate is performed, the aspirate should immediately be processed and cultured. Other techniques for aspiration (e.g. without injecting saline, or using ultrasounds) may be more efficacious.

## References

[CR1] Benoit SR, Ellingson KD, Waterman SH, Pearson ML (2014). Antimicrobial resistance in eight US hospitals along the US-Mexico border, 2000–2006. Epidemiol Infect.

[CR2] Edelsberg J, Weycker D, Barron R, Li X, Wu H, Oster G, Badre S, Langeberg WJ, Weber DJ (2014). Prevalence of antibiotic resistance in US hospitals. Diagn Microbiol Infect Dis.

[CR3] Epperly TD (1986). The value of needle aspiration in the management of cellulitis. J Fam Pract.

[CR4] Garau J, Blasi F, Medina J, McBride K, Ostermann H, REACH study group (2015) Early response to antibiotic treatment in European patients hospitalized with complicated skin and soft tissue infections: analysis of the REACH study. BMC Infect Dis 15:78-015-0822-2 10.1186/s12879-015-0822-2 PMC4352248 25879713

[CR5] Goldgeier MH (1983) The microbial evaluation of acute cellulitis. Cutis 31:649–650, 653–654, 656 6602690

[CR6] Gunderson CG, Martinello RA (2012). A systematic review of bacteremias in cellulitis and erysipelas. J Infect.

[CR7] Howe PM, Eduardo Fajardo J, Orcutt MA (1987). Etiologic diagnosis of cellulitis: comparison of aspirates obtained from the leading edge and the point of maximal inflammation. Pediatr Infect Dis J.

[CR8] Johnson AP (2011) Methicillin-resistant Staphylococcus aureus: the European landscape. J Antimicrob Chemother 66(Suppl 4):iv43–iv48 10.1093/jac/dkr076 21521706

[CR9] Lebre C, Girard-Pipau F, Roujeau JC, Revuz J, Saiag P, Chosidow O (1996). Value of fine-needle aspiration in infectious cellulitis. Arch Dermatol.

[CR10] Lee PC, Turnidge J, McDonald PJ (1985). Fine-needle aspiration biopsy in diagnosis of soft tissue infections. J Clin Microbiol.

[CR11] Mandell GL, Bennett JE, Dolin R (2010) Mandell, Douglas and Bennett’s principles and practice of infectious diseases, p 1307

[CR12] Moet GJ, Jones RN, Biedenbach DJ, Stilwell MG, Fritsche TR (2007). Contemporary causes of skin and soft tissue infections in North America, Latin America, and Europe: report from the SENTRY Antimicrobial Surveillance Program (1998–2004). Diagn Microbiol Infect Dis.

[CR13] Newell PM, Norden CW (1988). Value of needle aspiration in bacteriologic diagnosis of cellulitis in adults. J Clin Microbiol.

[CR14] Noh JY, Cheong HJ, Song JY, Hong SJ, Myung JS, Choi WS, Jo YM, Heo JY, Kim WJ (2011). Skin and soft tissue infections: experience over a five-year period and clinical usefulness of ultrasonography-guided gun biopsy-based culture. Scand J Infect Dis.

[CR15] Pallin DJ, Egan DJ, Pelletier AJ, Espinola JA, Hooper DC, Camargo CA (2008). Increased US emergency department visits for skin and soft tissue infections, and changes in antibiotic choices, during the emergence of community-associated methicillin-resistant Staphylococcus aureus. Ann Emerg Med.

[CR16] Patel Wylie F, Kaplan SL, Mason EO, Allen CH (2011). Needle aspiration for the etiologic diagnosis of children with cellulitis in the era of community-acquired methicillin-resistant Staphylococcus aureus. Clin Pediatr (Phila).

[CR17] Perl B, Gottehrer NP, Raveh D, Schlesinger Y, Rudensky B, Yinnon AM (1999). Cost-effectiveness of blood cultures for adult patients with cellulitis. Clin Infect Dis.

[CR18] Sachs MK (1990). The optimum use of needle aspiration in the bacteriologic diagnosis of cellulitis in adults. Arch Intern Med.

[CR19] Sigurdsson AF, Gudmundsson S (1989). The etiology of bacterial cellulitis as determined by fine-needle aspiration. Scand J Infect Dis.

[CR20] Weiss AJ, Wier LM, Stocks C, Blanchard J (2011) Overview of emergency department visits in the United States. http://www.hcup-us.ahrq.gov/reports/statbriefs/sb174-Emergency-Department-Visits-Overview.pdf 25144109

[CR21] Weiss J, Wier LM, Stocks C, Blanchard J (2014) Emergency departments (EDs) provide a significant source of http://www.hcup-us.ahrq.gov/reports/statbriefs/sb174-Emergency-Department-Visits-Overview.pdf

